# Impact and Implementation of Simulation-Based Training for Safety

**DOI:** 10.1155/2013/652956

**Published:** 2013-11-07

**Authors:** Federico F. Bilotta, Samantha M. Werner, Sergio D. Bergese, Giovanni Rosa

**Affiliations:** ^1^Department of Anesthesiology, University of Rome La Sapienza, Via Acherusio 16, 00199 Rome, Italy; ^2^Department of Anesthesiology, The Ohio State University Wexner Medical Center, N411 Doan Hall, 410 W. 10th Avenue, Columbus, OH 43210, USA; ^3^Department of Anesthesiology, University of Rome La Sapienza, Via Siracusa 12, 0016 Rome, Italy

## Abstract

Patient safety is an issue of imminent concern in the high-risk field of medicine, and systematic changes that alter the way medical professionals approach patient care are needed. Simulation-based training (SBT) is an exemplary solution for addressing the dynamic medical environment of today. Grounded in methodologies developed by the aviation industry, SBT exceeds traditional didactic and apprenticeship models in terms of speed of learning, amount of information retained, and capability for deliberate practice. SBT remains an option in many medical schools and continuing medical education curriculums (CMEs), though its use in training has been shown to improve clinical practice. Future simulation-based anesthesiology training research needs to develop methods for measuring both the degree to which training translates into increased practitioner competency and the effect of training on safety improvements for patients.

## 1. Introduction

Despite tremendous technological and societal improvements over the past several decades, healthcare, especially in the perioperative setting, remains among the most risky activities for a human being [1]. A comparison of ideal, ultra safe, regulated, and dangerous human systems in rates of error per operation lists the categories shown in Table 1 [2].

From this analysis, a hospital stay is as dangerous as bungee jumping and a traveler has a better chance of receiving luggage at a new destination than a prescribed medication postoperatively [2, 3]. In this paper, we review and explain the key components of simulator-based training scenarios that aim to increase perioperative safety.

The authors of this paper studied the current literature on simulation-based training for the practice of anesthesiologists based on the SBT anesthesiology program, CRM in aviation, and other healthcare and human management fields. An individual investigator performed a keyword search using the PubMed, Ohio State University Library network, and Google Scholar databases with the keywords including *anesthesiology, simulation training, CRM, ACRM, *and* human management*. Papers included as references were published between the years 1998 and 2013. A total of 33 papers with diverse experimental designs were used, including descriptive studies, reports, analyses of simulation equipment, and technique evaluations. The majority of the studies covered topics of simulation training for skill acquisition. Few studies addressed the long-term effects of simulation training and its translation to improvements in patient safety revealing a need for research that measures long-term outcomes.

## 2. Demand for Improvements in Patient Safety

One in every 150 patients admitted to a hospital dies as a consequence of an adverse event [4]. Interventions such as assigning surgical procedures to high-volume centers, establishing training programs for laparoscopic surgery, and improving the quality of teamwork in the operating room have been suggested as patient safety intervention strategies [5–7]. Yet, examples of hospitals systematically employing these solutions in practice are rare [8].

Healthcare regulators and administrators in addition to practitioners and patients are demanding drastic improvements in safety and care. The public's perceptions of safety are important and healthcare consumers voice the following concerns.

*Issues of Concern Regarding the Increasing Demands for Improvement*. Will financial pressures and organizational changes in healthcare [10]

- degrade practitioners' expertise?
- create conflicting goals and incentives?
- increase workloads?
- reduce safety margins?

*Questions to Be Addressed*. Further considerations [11] are as follows.

- How do we train physicians while protecting patients?
- How do we meet the needs of individual patients while still benefitting society?
- How do we reach financial goals while striving for patient safety?

## 3. Aviation Training

High-risk fields require intensive training that primes practitioners to handle challenging situations with ease. The aviation industry has weathered decades of safety challenges using a rigorous curriculum called simulation-based training or SBT. This method has been adapted for anesthesiology as well as other high-risk fields such as nuclear power, the military, and various medical fields including emergency and trauma medicine, intensive care, and cardiac arrest response teams [12, 13].

In order to maximize training safety and to minimize risk, aviation trainers have enhanced flight professionals' skills using crew resource management (CRM), a simulation-based training module designed for aviation crew members [14]. Instances of CRM simulators include virtual cockpit simulators and virtual reality parachute flight simulators that prepare smoke jumpers for forest fires [15].

It is interesting to note that the aviation industry as a whole moved from a safety rating of “risky” in the late 1950s to one of “safer” in a span of only several years. The robust safety improvements can be owed to increased aircraft reliability and a higher standard for training by means of simulation [14]. As a result of safety improvements, SBT is mandated and culturally accepted by pilots and pilots in training as a reliable and trustworthy educational tool [10].

The field of anesthesiology began using anesthesia crisis resource management (ACRM), a semblance of the aviation industry's crisis resource management (CRM) for emergency scenarios in the 1980s [16]. CRM is the epitome of simulation training in aviation. Its emphasis is decision-making and teamwork. The basis of the training is simulated crisis scenarios that are videotaped and then watched by team facilitators and participants in an comprehensive debriefing session [12].

## 4. Who to Blame: Human Error or System Malfunction?

Current lapses in care are influenced by many factors; a lack of emergency procedures and a missing system of training for nontechnical skills are among them [12]. Nontechnical skills, such as communication and teamwork, can be difficult to attain in real-life settings. In apprenticeship training, events are unpredictable and students spend much of this time as passive observers.

Additionally, practitioners often are unable and do not exemplify integration of technical and nontechnical skills, likely because they have not been taught themselves. Solutions are within reach and can be found in tasks such as creating an emergency procedure manual, developing a theory of dynamic decision-making for complexity, and using simulation crisis training in a safe environment with instructional feedback as a complement to current curriculums. When used as a complement to current teaching methods, a combination of SBT with classroom teaching offers the most viable solution to current gaps in medical education [12, 17].

Medical error can be explained upon the basis that people are unreliable components in an otherwise successful system. Practitioners directly interact with a hazardous process. Medical error can also be explained by expanding the pathway of error to include systematic vulnerabilities outside of practitioners' direct interactions. These systematic vulnerabilities that contribute to medical error include the regulators, administrators and policy makers who create demands for healthcare production. By recognizing that both direct and indirect forces contribute to medical malfunction, opportunities of failure can be transformed into opportunities for success.

## 5. Solutions for Practice

In a study of 44 final-year medical students at a medical school in Frankfurt, Germany, half of the students completed a SBT curriculum, while half were a part of a control group. The intervention group received simulation training based on basic life support, advanced cardiac life support, and advanced trauma life support over a three-day simulation training, while the control group attended three emergency department shifts in a shadowing role. The intervention group scored significantly higher than the control group on an objective structured clinical examination with a checklist rating. The intervention group scored 90% in a CPR situation, while the control group scored 62%. The lowest scoring scenario for both groups was a trauma reenactment in which the intervention group scored 76% and the control group scored 52% [18]. The results of the study were significant enough in showing the benefits of a standardized SBT curriculum for undergraduate medical students that the research training was integrated into the traditional course of study.

SBT can bridge the gaps in anesthetic practice by intensifying training through immediate clinical-simulated practice. The use of procedures, simplified and effective surgical procedure pathway checklists, investigation of second stories that delve into the systematic demands that ask healthcare professionals to produce more results in a shorter amount of time, creating cultures of safety that support SBT integration, and searching for specific ways of improving medical team communication will all contribute to improved patient safety outcomes and increased professional competency. These components are

- preanesthesia checklist,
- communication skills,
- procedural emergency management.

## 6. Preanesthesia Checklist

In a systematic review of anesthesia journals from 2001 to 2010, a total of 320 papers on the use of SBT were analyzed with 34% (110 papers) of the papers analyzing technical and nontechnical skill assessments by means of structured checklists [19]. Similarly, the joint commission, along with an abundance of international hospitals, supports the use of safety checklists to avoid wrong-site associated surgical incidents. Current statistics do not reflect drastic changes in rate of patient safety profiles and instead support the claim that checklists may “involve complexity without clear added benefit” [20]. Checklists may be ineffective if providers skip checklist steps or check off items that have not yet been performed.

The incidence, patterns, and prevention of wrong-site surgery show that of approximately 2.8 million surgeries scrutinized from the years of 1985–2004, one third of all errors began before the patient's arrival at the hospital on the day of surgery [20]. Properly used checklists have been implemented presurgically to address wrong-site surgery that occurs every 0.09–4.5 surgeries per 10,000 surgeries performed [9, 20].

Six intervention hospitals in The Netherlands implemented a surgical patient safety system (SURPASS) checklist from October 2007 to March 2009 [8]. The checklist served to follow the surgical pathway from admission to discharge. Its implementation at these hospitals over 6–9 months showed that complications per 100 patients decreased from 27.3 to 16.7 and that in-hospital mortality decreased from 1.5% to 0.8%. Decision-making that relies on the results of a simple and research-tested checklist can avoid cognitive errors, and ultimately, medical mistakes [21, 22]. Checklists with defined target end points can be used to define clinical scenarios into a flow-chart-like picture and to generate a decision-making process tailored to the recorded information.

## 7. Communication Skills

Ineffective communication is responsible for up to 70% of medical errors and inadvertent patient harm [23, 24]. Implementing simulator-based training for Intensive Care Unit (ICU) staff has effectively improved team communication skills [25]. In a study of 152 members of ICU staff at a Swedish hospital, participants were administered an interprofessional team training program that created a need to talk, specifically regarding complex care situations. Nurse self-reports revealed the program to be one of substantial value in addressing learned behaviors that can improve everyday work and contribute to better team collaboration. The study also recognized obstacles to successful systemic implementation of SBT [26] as follows:

- shortage of staff,
- overtime for staff,
- demands for hospital beds,
- budget cuts,
- segregated meetings for nurses and physicians (scheduling constraints).

These points must be addressed when implementing SBT curriculum as a sustainable, long-term solution for improving patient safety and increasing medical professional competency.

## 8. Procedural Emergency Management

Occasionally, anesthesiologists may encounter uncommon clinical scenarios, such as cerebral aneurysm rupture during delivery. Minimal postcertification exposure to uncommon events leads to inappropriate management when those complications do occur. More adequate training for rare medical encounters that includes simulator-based training integrated into the standardized medical education curriculum can provide necessary basic-skill training.

A mobile Intensive Care Unit (MICU) implemented by the mandate of the Dutch Ministry of Public Health in 2007 administered crisis resource management training to intensivists, ICU nurses, and the MICU vehicle drivers. The training identified potential problems such as failing to ask for intubation of a respiratory-compromised patient at intake, late responses to alarms of the ventilator, perfusor pump, or monitor and not anticipating a possible shortage of medication [27]. Identification and addressing these specific problems in training increased the likelihood that medical staff possessed the skills to address them in reality.

## 9. Components of Successful Simulation

SBT is characterized by feedback, repetition, variations in degree of difficulty, use in a controlled environment, and defined outcomes for measureable learning [11]. It provides the support for an instructional process that substitutes real patient encounters with artificial models, live actors, or virtual reality [12]. Learning is accelerated when participants are given opportunities to alter their clinically simulated approach immediately in response to constructive criticism, retain information through repeated practice, encounter situations in increasing levels of difficulty, and aim to achieve clear goals such as those outlined in a module checklist.

Each simulation team consists of a different medical discipline such as anesthesiologists, nurses, or surgeons. During SBT, each crew comes together to work as a team in a scenario lasting 25–40 minutes. Cross-disciplinary training is performed by rotating members into various roles during scenarios. Through this rotation, each role will gain a comprehensive perspective of key tasks that need to be performed in that specific role in order to be a successful member of a medical team [12].

Debriefing, offering constructive and immediate feedback, remains the most important factor that contributes to improved learning and skill retention [11, 12, 16]. It constitutes SBT as *training* rather than simply simulation. Without timely and appropriate feedback, trainees cannot learn from mistakes and successes. Then, the trainee can adjust strategies and improve competencies while proceeding through the simulation [28, 29]. The benefits of simulation training are wide-ranging and include safe and deliberate practice without causing harm to real-life patients, the acquisition of nontechnical skills such as team efficiency in a surgical setting, accommodation to multiple learning strategies, and the existence of measurable outcomes [30].

## 10. Does SBT Translate into Improvements in Patient Safety?

Little testing has been performed, yet limited research shows that using simulation as a teaching methodology does indeed transfer to improvements in patient care [31]. Posttest scores of senior anesthesia trainees who received simulation training for weaning from cardiopulmonary bypass performed better in the real life situation than those who received traditional interactive seminars alone [31]. The posttest score for the simulation-trained group was 89.9%, while the traditionally trained group averaged 75.4%. Retention tests five weeks after training were more drastic with the simulation group scoring 93.2% and the traditional seminar trained group scoring 77%. Score values for nontechnical skill evaluation were 14.1 for the simulation group and 11.7 for those trained by means of traditional seminars. Thus, the impact of simulation training in real life is measureable.

There is minimal research examining performance transfer, sustainability, or direct patient outcomes and experiences. Only 10% of 320 reviewed papers on SBT included data from the clinical environment, a necessary component for examining patient outcomes [19]. Future research must consider whether or not SBT translates into improvements in practitioner competency and patient care by measuring significant improvements [18, 32]. Researchers claim that it is difficult, if not logistically impossible, to assess patient outcomes, and the impact of SBT on practitioner performance and ability still remains to be measured. Items to be measured include behavior performance by means of communication, leadership, fellowship, distribution of workload, and overall CRM performance. No standardized scoring system yet exists [12].

## 11. Limitations

Although there is no substitute for work with real patients, SBT is a worthy addition to medical education. In terms of situational exposure to what may happen in a particular emergency and what steps to follow in order to claim a successful outcome, SBT is exceptional. However, when comparing SBT mannequins to real-life humans, SBT is not always accurate. In a study of 20 adult trauma patients without head or neck injuries in comparison to four high fidelity patient simulators, computed tomography scans measured radiographic measurements. Results showed that the volume of the pharyngeal airspace differed significantly between actual patients and simulators. The average pharyngeal airspace in patients was 13.5 cm^3^ and significantly larger in the mannequins: SimMan 68.5 cm^3^, SimMan 3G 35.4 cm^3^, HPS 30.6 cm^3^, HAL 40.1 cm^3^, and Laerdal Manikin 65.9 cm^3^ [33]. A solution is to create simulators that more closely match actual patients, are high-fidelity, and can contribute to improved competency in airway management training. However, analysts of simulators must also consider that more spacious SimMan airways may provide for beneficial introductory training that actual patients could not supply.

Other limitations in the use of SBT are an expensive initial cost for equipment and facilitator training, a lack of rigorous proof of effect in translational science, and a strong resistance to change amongst health professionals [13]. The need for dedicated simulation rooms with audio-video systems may prohibit institutions without these resources from using simulation-based techniques [34]. Furthermore, simulation training may be predisposed to a subjective bias if facilitators are not sufficiently trained [16].

## 12. Conclusion

In this paper, we have presented how and why simulator-based training should be implemented into residency training and continuing medical education to increase perioperative safety by means of improvements in communication skill, use of a checklist, and procedural emergency management. These themes echo those found in civil aviation training that has experienced much success in reaching high standards of safety and serve to develop dedicated programs such as ACRM. Once implemented, anesthesiology can serve a model for SBT curriculum integration for other medical fields [12]. Increasing medical accuracy by improving skilled professional performance through SBT translates into benefits for society at large [35].

The future of simulation-based education is as a modality integrated into traditional medical school curriculums and offered for practicing anesthesiologists in the form of CME credit. Professional training must concurrently address management of standard clinical practice, prevention of complications, and management of unusual clinical scenarios through integration of SBT into standardized medical education and CME.

## Figures and Tables

**Table 1:** Comparison of safety of different human activities [9].

Ultra safe	<1/100,000 Deaths per year	Scheduled airlines, nuclear power, european railroads, aircraft carriers
Regulated		Car driving, chemical industry, charter flights
Dangerous	>1/1000 Deaths per year	Bungee jumping, extreme mountain climbing, motorcycle racing, hospital stays
